# Using geospatial models to map zero-dose children: factors associated with zero-dose vaccination status before and after a mass measles and rubella vaccination campaign in Southern province, Zambia

**DOI:** 10.1136/bmjgh-2021-007479

**Published:** 2021-12-30

**Authors:** Rohan Arambepola, Yangyupei Yang, Kyle Hutchinson, Francis Dien Mwansa, Julie Ann Doherty, Frazer Bwalya, Phillimon Ndubani, Gloria Musukwa, William John Moss, Amy Wesolowski, Simon Mutembo

**Affiliations:** 1 Epidemiology, Johns Hopkins University Bloomberg School of Public Health, Baltimore, Maryland, USA; 2 International Vaccine Access Center, International Health, Johns Hopkins University Bloomberg School of Public Health, Baltimore, Maryland, USA; 3 Implementation Science, Akros Zamba, Lusaka, Zambia; 4 Directorate of Public Health and Research, Zambia Ministry of Health, Lusaka, Zambia; 5 Administration, Macha Research Trust, Choma, Zambia; 6 Choma General Hospital, Zambia Ministry of Health, Lusaka, Zambia

**Keywords:** measles, immunisation, mathematical modelling, epidemiology

## Abstract

**Introduction:**

Despite gains in global coverage of childhood vaccines, many children remain undervaccinated. Although mass vaccination campaigns are commonly conducted to reach these children their effectiveness is unclear. We evaluated the effectiveness of a mass vaccination campaign in reaching zero-dose children.

**Methods:**

We conducted a prospective study in 10 health centre catchment areas in Southern province, Zambia in November 2020. About 2 months before a national mass measles and rubella vaccination campaign conducted by the Ministry of Health, we used aerial satellite maps to identify built structures. These structures were visited and diphtheria-tetanus-pertussis (DTP) and measles zero-dose children were identified (children who had not received any DTP or measles-containing vaccines, respectively). After the campaign, households where measles zero-dose children were previously identified were targeted for mop-up vaccination and to assess if these children were vaccinated during the campaign. A Bayesian geospatial model was used to identify factors associated with zero-dose status and measles zero-dose children being reached during the campaign. We also produced fine-scale zero-dose prevalence maps and identified optimal locations for additional vaccination sites.

**Results:**

Before the vaccination campaign, 17.3% of children under 9 months were DTP zero-dose and 4.3% of children 9–60 months were measles zero-dose. Of the 461 measles zero-dose children identified before the vaccination campaign, 338 (73.3%) were vaccinated during the campaign and 118 (25.6%) were reached by a targeted mop-up activity. The presence of other children in the household, younger age, greater travel time to health facilities and living between health facility catchment areas were associated with zero-dose status. Mapping zero-dose prevalence revealed substantial heterogeneity within and between catchment areas. Several potential locations were identified for additional vaccination sites.

**Conclusion:**

Fine-scale variation in zero-dose prevalence and the impact of accessibility to healthcare facilities on vaccination coverage were identified. Geospatial modelling can aid targeted vaccination activities.

**Key questions**

**What is already known?**

- In many low-income and lower-middle income countries, improvements in routine childhood vaccination coverage have stalled.
- An estimated 17 million children globally have not received any routine vaccinations (zero-dose children).
- Zero-dose children, and those who have not received any doses of specific vaccines such as measles, remain vulnerable to preventable diseases and can sustain transmission in otherwise highly vaccinated populations.
- A lack of understanding of the number and spatial distribution of zero-dose children make targeting vaccination activities to reach this group challenging.

**What are the new findings?**

- Prior to a mass measles and rubella vaccination campaign, 17% of children younger than 9 months of age in the study area had not received the diphtheria-tetanus-pertussis vaccine and 4% of children between 9 and 60 months of age had not received a measles-containing vaccine.
- Over a quarter of the children identified as not having received a measles-containing vaccine before the measles and rubella mass vaccination campaign were not vaccinated during the campaign.
- Geospatial models revealed substantial fine-scale variation in zero-dose status and accurately predicted optimal locations for additional vaccination sites.

**Key questions**

**What do the new findings imply?**

- Despite high overall estimates of campaign coverage, campaigns may still not be vaccinating all eligible children.
- There is potential for using similar household-level geospatial survey and modelling strategies to improve targeting of vaccination activities to reach zero-dose children.

## Introduction

Significant gains in vaccination coverage have been made globally since the 1980s due to substantial investment in childhood immunisation services. However, in the past decade, progress has stagnated and routine vaccination coverage has declined in many countries.^1 2^ Widespread disruption to vaccine delivery due to the COVID-19 pandemic has compounded this problem, with global coverage of the first dose of a diphtheria-tetanus-pertussis-containing vaccine (DTP1) and first dose of a measles-containing vaccine (MCV1) falling from 90% and 86% in 2019 to 87% and 84% in 2020, respectively.^3 4^ This stalling progress and disruption to routine vaccination activities has resulted in pockets of unvaccinated and undervaccinated communities. Communities where vaccination rates are below herd immunity thresholds are at risk of outbreaks of vaccine preventable diseases.^5–7^

Communities at risk of outbreaks are comprised of children who missed some vaccine doses as well as children who did not receive any routine vaccinations, the latter referred to as zero-dose children. In practice, zero-dose children are often defined as children who have not received a DTP1 vaccine.^8–10^ There were an estimated 17 million zero-dose children in 2020, the majority living in sub-Saharan Africa or conflict-affected areas.^4 9 11 12^ Research on the ‘immunisation cascade’, which describes how children move from zero-dose to fully vaccinated, suggests that in many Low and Middle Income Countries (LMICs), vaccine coverage is polarised, with most children either receiving all or almost all vaccines, or few to none.^13^

Coverage of routine vaccinations in Zambia is generally high, with an estimated 94%, 88% and 93% of eligible children having received DTP1, DTP3 and MCV1 vaccinations, respectively, although coverage of MCV2 lags far behind at 66%.^2^ However, these overall coverage values cannot inform fine-scale heterogeneity in vaccination rates and the remaining unvaccinated and undervaccinated children may be at risk, particularly those living in communities with below-average vaccination coverage where sustained transmission is more likely. In Zambia, as in many LMICs, mass measles and rubella vaccination campaigns are carried out with the aim of vaccinating children who have not received their routine doses. These nationwide non-selective campaigns are carried out by the Ministry of Health in Zambia every 4 years to avoid the accumulation of birth cohorts of susceptible children as part of the measles elimination strategy. Understanding the prevalence and spatial distribution of children in the community who have received few vaccines or are zero-dose is challenging, as these children are likely to be less engaged with routine healthcare services.^14^ Lack of data on the location of zero-dose children and an incomplete understanding of the barriers to vaccinations make targeting of intensified vaccination activities challenging. Additionally, for both routine and mass vaccination campaigns, it is difficult to determine if underserved communities were reached.

Several studies have investigated risk factors for low vaccine coverage or incomplete vaccination, often identifying accessibility to healthcare, maternal education, parental attitude and socioeconomic status as important factors.^15–20^ While there has been limited work in this area in Zambia in particular, Setse *et al*^21^ found that lower levels of maternal education and larger family sizes were associated with incomplete DTP and polio vaccination in Lusaka, Zambia. However, remote or marginalised communities may be under-represented in studies that recruit participants from those already engaged with the formal healthcare system, for example, studies requiring child health cards for eligibility.^15 16^ Furthermore, in many low-income and middle-income countries, improvements in overall vaccination coverage and reductions in zero-dose prevalence do not coincide, suggesting that specific intervention strategies are needed to reach zero-dose children.^20^ Post campaign coverage surveys may be used to identify communities not reached or factors impacting campaign coverage, but these surveys have limited spatial and demographic detail and may also suffer from selection bias.^22^ Moreover, it is unclear how to operationalise the results of these studies on vaccination status or campaign coverage to improve the effectiveness of vaccination activities. Data and models that can be used to locate communities with a high prevalence of zero-dose children may help programmes better allocate existing services. However, data from health facilities or number of doses given in a campaign cannot capture fine-scale spatial heterogeneities in coverage and are unlikely to be representative.

Detailed household mapping of eligible and vaccinated children pre and post a national vaccination campaign can provide more information on the distribution of zero-dose children and which of these children are reached by the campaign. These data also provide a framework to model the effectiveness of various campaign strategies, including location of outreach vaccine sites. Here we describe mapping and vaccination activities carried out in parts of Choma District, Southern Province, Zambia. These activities took place before and after a national mass measles and rubella vaccination that was conducted in November 2020 by the Ministry of Health. Prior to the campaign, households were enumerated and the DTP or measles vaccination status of children under 5 years of age was recorded. After the campaign, households with unvaccinated and undervaccinated children where revisited and whether the children eligible for the mass vaccination campaign had been vaccinated in the campaign was recorded. Any children remaining unvaccinated or undervaccinated for DTP, measles and other routine vaccinations were offered these vaccinations. Using these data, we developed a geospatial model to estimate fine-scale heterogeneity in DTP and measles (given the higher critical vaccination threshold) zero-dose prevalence. We also developed a geospatial model to estimate the fine-scale variation in the probability of a measles zero-dose child being vaccinated in the mass campaign. Finally, we used the latter model to predict the effect of adding new vaccination sites in different locations on the number of zero-dose children reached in a vaccination campaign. Although the mass vaccination campaign conducted by the Ministry of Health was a measles and rubella campaign, information on DTP vaccination was collected to gain a better understanding of zero-dose communities at different age groups and use these data to understand the utility of the model when applied to coverage of different vaccine antigens.

## Methods

### Study design and population

**Figure 1:**
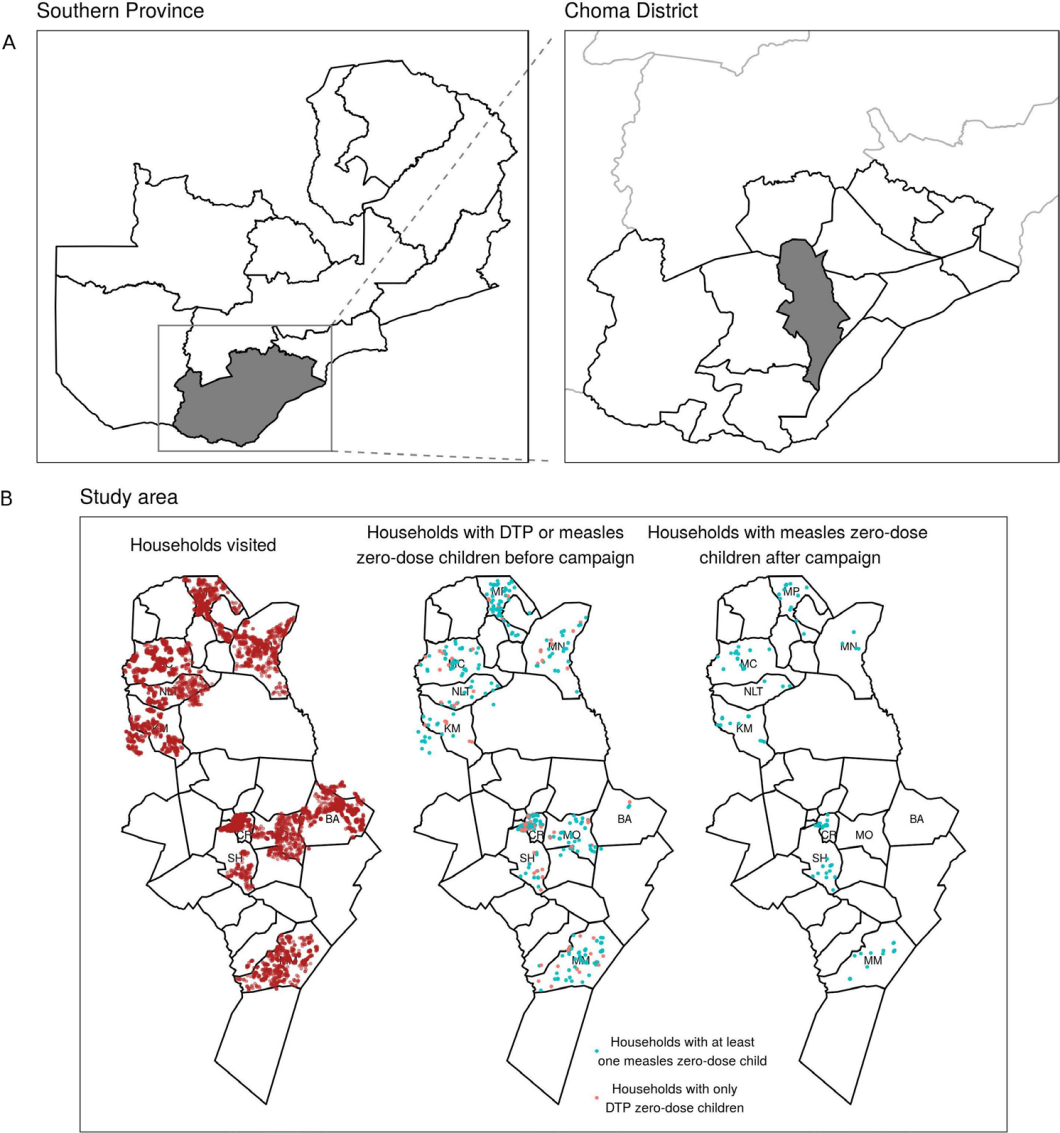
Study area. (A) Location of Choma District within Zambia and Southern Province. (B) Health facility catchment areas in Choma District with the 10 catchment areas in this study labelled: Batoka (BA), Choma Railway Surgery (CR), Kamwanu (KM), Macha (MC), Masuku Mission (MM), Mangunza (MN), Mapanza (MP), Nalituba (NLT), Shampande (SH) and Mochipapa (MO). The points represent the locations of all households visited (left), households containing zero-dose children before the campaign (middle, shaded in blue for households with any measles zero-dose children) and households containing measles zero-dose children that were not vaccinated during the campaign (right). DTP, diphtheria-tetanus-pertussis.

The study was conducted in 10 health facility catchment areas of Choma District, Southern Province, Zambia (figure 1). Two of the catchment areas (Choma Railway Surgery and Shampande) are densely populated urban settings. The remaining catchment areas are rural areas populated by subsistence farmers living in scattered homesteads, characteristic of much of rural sub-Saharan Africa.^23^ Overall, reported vaccine coverage in Choma District is high (DTP1=99%, MCV1=93% and MCV2=73% in 2020).^24^

We conducted a prospective study to quantify the vaccination status of children in the study area before and after a nationwide mass measles and rubella vaccination campaign. The campaign was carried out by the Ministry of Health between 20 and 29 November 2020. All children in Zambia between 9 and 60 months of age were eligible for the mass measles and rubella vaccination campaign, regardless of previous vaccination history.

Approximately 6 weeks prior to the campaign, all structures identified in the study area using satellite imagery were visited by community health volunteers (CHVs) and eligible children were registered. All built structures in the health centre catchment areas were identified by satellite imagery and the structures were divided into operational zones for CHVs. The community volunteers used these maps to navigate the zones and registered household structures. These structures included permanent and temporary housing. Every child younger than 60 months who lived in a household within the study the study area was eligible for the study. Vaccination status was recorded based on the child’s age. For children under 9 months of age, DTP1 vaccination was recorded. For children between 9 and 60 months of age, measles and rubella vaccination status was recorded. The age and sex of each child was also recorded. For the purposes of this study, a child was defined as DTP zero-dose if they were younger than 9 months of age and had not received DTP1, and measles zero-dose if they were between 9 and 60 months of age and had not received any dose of a MCV. We did not collect information on DTP1 for children over 9 months. Here, zero-dose status refers to both groups.

One week after the vaccination campaign, households where measles zero-dose children were identified were revisited to see if these children were vaccinated during the campaign. If a child was not vaccinated during the campaign, a measles and rubella dose was offered. Other vaccinations were also offered during these mop-up activities: (1) If a child in these households was under 9 months of age but had not received a DTP1 vaccination, they were offered a vaccination, and (2) if any child in these or nearby households was found to be missing any routine vaccine for their age, these vaccinations were offered. For the purpose of our study, only information on DTP1 and measles and rubella vaccination were collected on our data collection tools. Information on measles, DTP1 and other vaccinations carried out by the CHVs was collected and added to the Ministry of Health records, so these children could be included in planning for future vaccination activities and routine services. No incentives for vaccination were provided.

### Data collection

Data were collected on electronic tablets using Reveal, an open-source platform for mapping populations and monitoring coverage of health interventions at household level.^25^ Manual and automated enumeration methods and algorithms were applied to high resolution satellite imagery to identify structures within the 10 health facility catchment areas. The operational health facility catchment area boundaries and major landmarks were mapped in a participatory manner with health facility staff and community health workers using google earth. These data were uploaded to the mobile component of the Reveal platform in the form of digital base-maps that assisted field teams with real-time navigation capabilities, to ensure all structures within a given area were identified and reached. Teams of CHVs, using Reveal’s mobile, map-based interface, then went door-to-door to obtain informed consent and register children eligible for the upcoming vaccination campaign. Information on vaccination status was collected based on vaccination cards, if available, and otherwise based on caregiver’s recall. After the campaign, teams of CHVs and health centre nurses used the Reveal interface to navigate back to the houses where measles zero-dose children were identified before the campaign to verify whether these children were vaccinated during the campaign.

CHVs conducted the household surveys supervised by environmental health technicians from the health centres. CHVs were recruited from among the CHVs who were already involved in other health centre activities and had a high school level of education. The Reveal platform was chosen as it allowed for smooth integration of the satellite image-based structure identification, navigation to these structures and data collection. This tool had previously been used for the implementation of indoor residual spraying for malaria in Zambia.^26 27^

### Data analysis

#### Univariate analyses

The relationships between several factors and zero-dose status before the mass vaccination campaign were initially assessed in univariate analyses. These factors were age, travel time to the nearest health facility, the presence of at least one other eligible child in the household and whether the household was ‘between health facilities’. Travel time, rather than distance, to the nearest health facility was used as this is a more meaningful metric of how accessible the nearest health facility was. Travel times were calculated using a friction surface^28^ based on walking speeds (see online supplemental material section 1 for more details). While a recent study found that friction surface-derived motorised travel times often underestimated true travel times in Nigeria,^29^ many of the potential reasons for this (such as high volumes of traffic at certain times of the day) are unlikely to apply to walking times in our largely rural study area. Furthermore, the friction-derived and true travel times showed strong correlation and therefore the strengths of associations between travel times and other variables would be similar using either values. A household was defined as being between health facilities if travel time to the second closest health facility was within 20% of the travel time to the nearest health facility. The location of these between facility households is shown in online supplemental figure 1 and this percentage was varied in a sensitivity analysis also detailed in supplementary material section 2. The relationship between the likelihood of a measles zero-dose child being vaccinated in the campaign and travel time to the nearest vaccination site was also investigated.

10.1136/bmjgh-2021-007479.supp1 Supplementary data

### Geostatistical model

Separate Bayesian geospatial models, based on the geostatistics framework pioneered by Diggle and others,^30–32^ were used to model: (1) DTP zero-dose prevalence, (2) measles zero-dose prevalence before the campaign and (3) probability of a measles zero-dose child being vaccinated during the campaign across the study area. The first two of these models used data from the precampaign household surveys, while the latter used data from both before and after the campaign. The DTP and measles zero-dose prevalence models are described here, while the latter model is described in the following section. These geostatistical models are multivariable models and therefore the effects of the different explanatory variables are considered together.

Let $y_{i}$ be the missing vaccination status of child i(i=1,...,1870forDTPandi=1,...,11649formeasles), that is $y_{i}$ was 1 if the child had not received DTP1 (if under 9 months) or MCV1 (if 9 months and older) and 0 if they had. Let $l_{i}$ be the location of the household this child lived in. Missing vaccination status was modelled as a realisation of a Bernoulli process,

$$y_{i}∼Bernoulli(p_{i})$$

with underlying probability $p_{i}$ for a child of this age at this location. This underlying probability was on a logit-scale as the sum of a linear contribution from covariates and a Gaussian process over space

$$logit(p_{i})=\beta_{0}+\beta^{T}X_{i}+f(l_{i})+\epsilon_{h_{i}}$$

Here $X_{i}$ were covariate values, f was the Gaussian process term and $\beta_{0},\beta$ were parameters to be learnt. The covariates were age, travel time to the nearest health facility (in minutes), presence of at least one older eligible child in the same household, presence of at least one younger eligible child in the same household, whether the household was between facilities and which health facility catchment area the household was in. The Gaussian process term accounted for spatial variation driven by unobserved factors and was given a Matern covariance structure parameterised by the range, ρ, and marginal variance, $\sigma^{2}$. A household-level random effect, $\epsilon_{h_{i}}$, was also included (where $h_{i}$ was the household that child i lived in) to account for repeated sampling from households containing multiple eligible children.

The Bayesian model was completed by placing priors of the model parameters. Normal priors were placed on $\beta_{0}$ and β with mean 0 and SD 1. Penalised complexity priors were used for the Mátern covariance parameters.^33^

To map predicted zero-dose prevalence across the study area, each model was refit with only spatial covariates included (ie, travel time to the nearest health facility and whether the location was between facilities).

### Effectiveness of additional outreach vaccination sites

The mass measles and rubella vaccination campaign carried out by the Ministry of Health was conducted at health facilities and temporary outreach vaccination sites set up across Choma District. We evaluated the effect of adding additional outreach vaccination sites in future campaigns. First a geostatistical model was fit to model the probability of a measles zero-dose child identified in the precampaign enumeration being vaccinated during the mass vaccination campaign. This model structure was the same as the zero-dose prevalence model previously described, with a child being vaccinated in the campaign modelled as a Bernouilli process based on an underlying probability. This probability was modelled as the sum of a linear combination of covariates and a Gaussian process term, again as in the zero-dose prevalence model. Here the covariates were age and travel time to the nearest campaign site. Campaign sites were defined as health facilities or outreach vaccination sites. After fitting the model, the probability of a measles zero-dose child of a given age and location being vaccinated in the campaign could be calculated. Let $p\left(a,l\right)$ be this probability for a child of a months of age at location l.

To evaluate the effect of adding an outreach vaccination site at a given location s, travel times to the nearest campaign site were recalculated with this additional site included. The fitted relationship was then used to calculate an updated probability, Missing argument for \hat), of a measles zero-dose child of a given age at a given location being vaccinated in the campaign. We assumed that individuals would not travel more than 60 min to be vaccinated and hence, the final probability of a child being vaccinated given this additional vaccination site was defined to be qa,l,s=q^a,l,s if l and s were within 60 min travel time of each other and $q\left(a,l,s\right)=p\left(a,l,s\right)$ otherwise.

The overall effect of adding this outreach vaccination site, $Eff\left(s\right)$, was defined as the difference between these probabilities, summed over all measles zero-dose children identified in the survey,

$$Eff\left(s\right)=\sum_{i=1}^{N}q\left(a_{i},l_{i},s\right)-p\left(a_{i},l_{i}\right).$$

In other words, the total effectiveness of an additional site was defined as the increase in vaccination probability of a child of a given age and location integrated over the empirical age and spatial distribution of measles zero-dose children. The effect of adding multiple additional outreach vaccination sites at different locations was similarly calculated by recalculating the travel times and vaccination probabilities.

An alternative form of the geospatial model for vaccination probability was also fit in which time to the nearest health facility and time to the nearest outreach vaccination site were included as separate covariates to investigate the sensitivity of our results to combining both (as time to the nearest campaign site) in the main analysis.

### Model validation

Both geostatistical models were validated using k-fold cross-validation. Evaluating model performance by at a household level is challenging due to high sampling variance of the data generating process at low prevalence values and may not be the most relevant metric from an operational perspective, as even targeted activities are unlikely to be planned at this level. A more relevant spatial scale is performance for small clusters of households, which we term settlements, that are used for planning of other public health measures in Zambia such as indoor residual spraying for malaria control.^34 35^ Observed and predicted prevalence was compared at the settlement level, where these settlements were groups of households generated by k-means spatial clustering, intended to approximate the true settlements in the study area. The number of households per cluster and the number of folds for k-fold cross-validation was varied (see online supplemental information section 3.1). We also investigated the potential for using the zero-dose prevalence model to predict prevalence in new locations by fitting the model on data from three catchment areas and making predictions for all other catchments.

An assumption underlying our analysis of the effect of adding outreach vaccination sites in different locations was that the learnt relationship between travel time to the nearest campaign site and probability of a measles zero-dose child being vaccinated in the campaign reflected the causal effect of campaign sites. If this relationship was confounded, however, this may not necessarily be the case. For example, if some unobserved factors (such as accessibility) caused outreach vaccination sites to be located in areas where children were already more likely to be reached by the campaign, this could inflate the apparent effect of outreach vaccination sites. A negative control was used to check whether such confounding existed.^36^ A negative control is a response variable that is similar to the response variable of interest (in this case whether a measles zero-dose child was vaccinated during the campaign) but which is known or believed to be unaffected by the explanatory variable (in this case distance to the nearest campaign site). The analysis is then repeated with this alternative response variable and if the learnt relationship with the explanatory variable is non-zero, then it suggests this analysis is confounded, and therefore the main analysis may also be confounded. We used measles zero-dose status before the campaign, which clearly could not be affected by the campaign itself, as a negative control to investigate any potential confounding.

## Results

**Figure 2:**
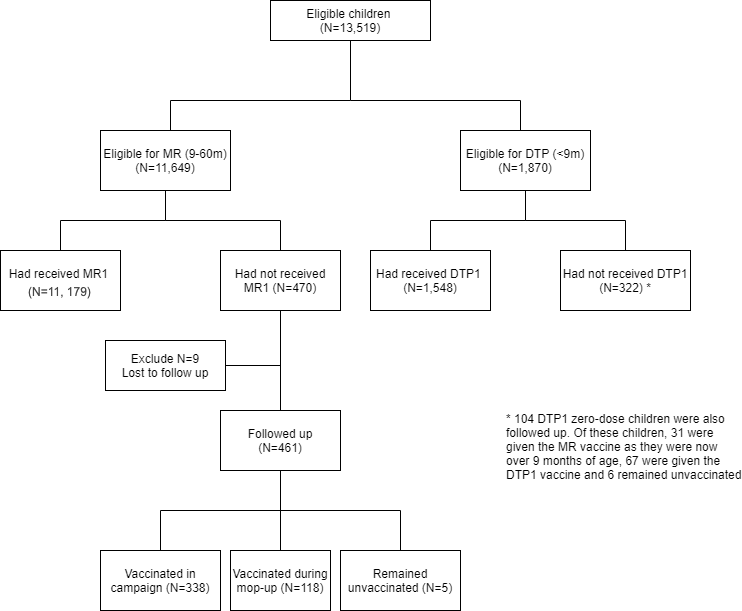
Flow chart describing the data collected. DTP, diphtheria-tetanus-pertussis.

In total, 41 952 structures were identified within the study area from aerial satellite imagery. Of these, 10 758 households were eligible for the study (with many households consisting of multiple structures). In the precampaign enumeration phase, 13 519 children were registered and eligible for the study, of whom 1870 (13.8%) were younger than 9 months and 11 649 (86.2%) were 9–60 months old (figure 2). Of the children younger than 9 months, 322 (17.3%) had not received DTP1 and were therefore classified as DTP zero-dose children. Four hundred and seventy (4.3%) children 9–60 months of age had not received MCV1 and were classified as measles zero-dose.

Approximately 1 week after the mass measles and rubella vaccination campaign, 461 of the measles zero-dose children identified in the precampaign enumeration were successfully followed up as part of a targeted mop-up activity. Of these, 338 (73.3%) received the measles and rubella vaccine during the campaign, 118 (25.6%) were vaccinated during this mop-up activity and 5 (1.1%) remained unvaccinated. During this mop-up activities, some of DTP zero-dose children identified in the initial survey were also followed-up and some additional children were registered, although this was not the focus of the CHVs and was carried out in a non-systematic way, detailed in online supplemental material section 4.

There was substantial heterogeneity in DTP and measles zero-dose prevalence prior to the mass vaccination campaign at the health facility catchment-level (table 1). The lowest prevalence for both was in Batoka (DTP1=7.1% and MCV1=0.2%), with the highest DTP zero-dose prevalence in Kamwanu (58.9%) and measles zero-dose prevalence in Mapanza (8.2%). There were no clear long-range spatial trends (eg, north to south) in DTP or measles zero-dose prevalence across the study area. At the subcatchment level, there was some evidence of spatial clustering only in Shampande and Mapanza catchment areas (Moran’s I, p<0.01, online supplemental material section 5).

**Table 1:** Observed DTP and measles zero-dose prevalence before the measles and rubella vaccination campaign by catchment area

Catchment	DTP zero-dose prevalence (%)	Measles zero-dose prevalence (%)
Batoka	7.1	0.2
Choma Railway Surgery	18.6	4.7
Kamwanu	58.9	3.7
Macha	15.6	3.7
Mangunza	15.2	3.6
Mapanza	14.3	8.2
Masuku Mission	21.7	7.2
Mochipapa	21.3	5.7
Nalituba	18.8	2.4
Shampande	14.8	3.1

Table 1: There was variation in zero dose prevalence across health facility catchment areas. The highest zero dose prevalence based on DTP was in the catchment area around Kamwanu rural health center.

DTP, diphtheria-tetanus-pertussis.

**Figure 3:**
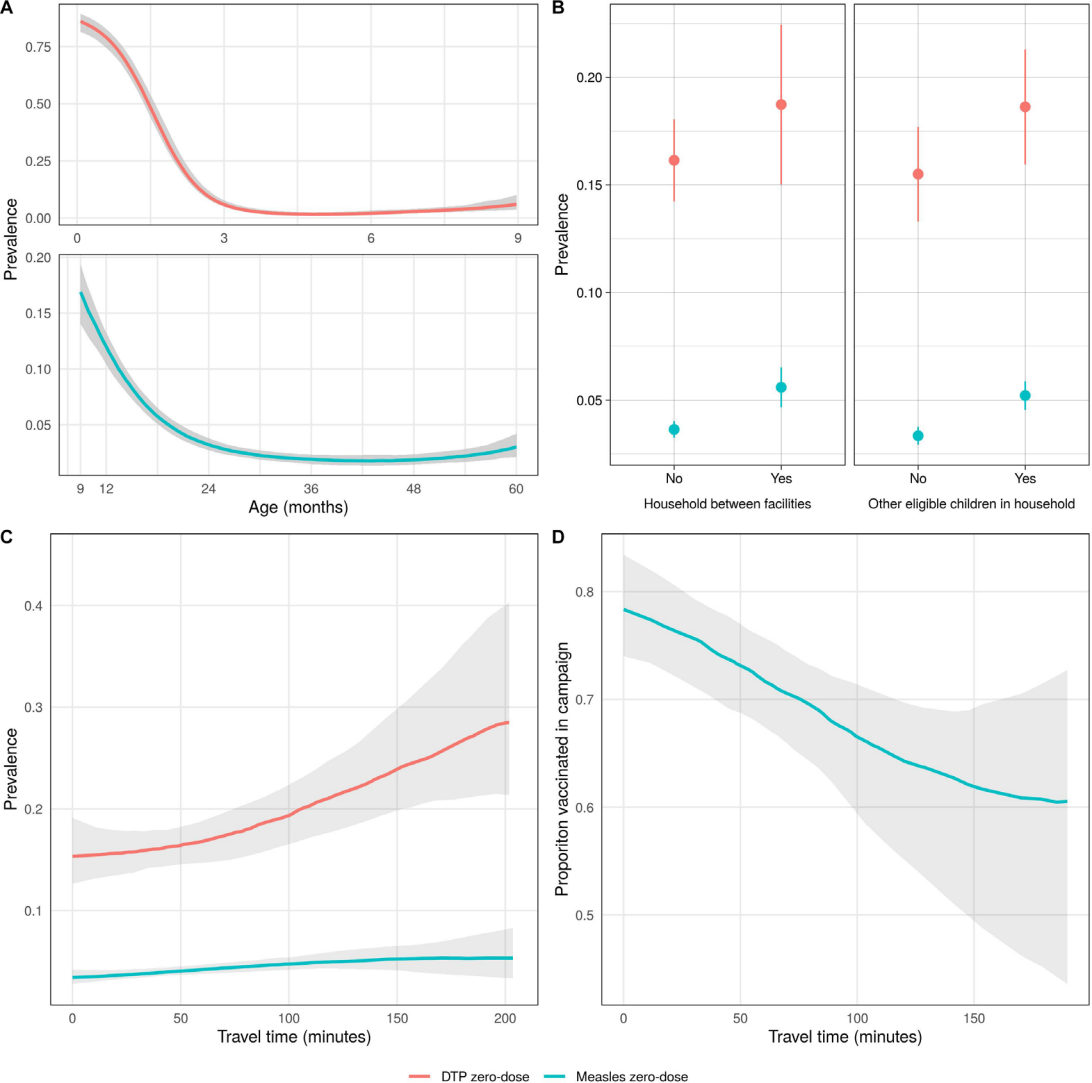
Univariate relationships between DTP and measles zero-dose prevalence and different factors with 95% credible intervals. For continuous variables (A, C, D), a non-parametric model was fit (see Supplementary material section 6). (A) DTP and measles zero-dose prevalence before vaccination campaign by age, (B) DTP and measles zero-dose prevalence before vaccination campaign by whether a child lived in a household between facilities or containing at least one other eligible child, (C) zero-dose and measles zero-dose prevalence before vaccination campaign by travel time to the nearest health facility and (D) probability of a measles zero-dose child being vaccinated during the campaign by travel time to the nearest campaign site. DTP, diphtheria-tetanus-pertussis.

Both DTP and measles zero-dose prevalence prior to the mass vaccination campaign decreased as age increased, and initially increased as travel time to the nearest health facility increased (figure 3A, C). As travel time increased further, DTP zero-dose prevalence increased but measles zero-dose prevalence plateaued, although in both cases, there was substantial uncertainty at these distances due to small numbers of observations. DTP and measles zero-dose prevalence was higher in children living in a household between facilities or with other eligible children, with both effects stronger for measles zero-dose status. The proportion of measles zero-dose children vaccinated during the mass vaccination campaign decreased as travel time to the nearest vaccination site increased (figure 3D).

When the joint relationship between these factors and DTP zero-dose prevalence was investigated using the geostatistical model, we found broadly similar results to the univariate analyses. An increase in age and the presence of at least one younger eligible child in the household were associated with increased DTP zero-dose prevalence (table 2). The effects of the other covariates were uncertain, with the 95% credible intervals containing zero (table 2). The magnitude of coefficient for age was particularly large, reflecting the initial steep decline in DTP zero-dose prevalence with age (figure 3). There was no clear association between most health facility catchment areas and DTP zero-dose prevalence except for Kamwanu and Nalituba, which were associated with increased and decreased prevalence, respectively.

**Table 2:** Mean and 95% credible interval for covariate effects in the DTP and measles zero-dose prevalence models

Covariate	Mean (CI)	
	DTP zero-dose	Measles zero-dose
Time to nearest facility	0.12 (−0.21 to 0.45)	0.14 (0.01 to 0.28)
Between facilities	−0.08 (−0.58 to 0.41)	0.21 (−0.05 to 0.46)
Age	−3.31 (−3.7 to 2.91)	−0.83 (−0.96 to 0.71)
Other older child in household	−0.07 (−0.43 to 0.29)	0.3 (0.08 to 0.51)
Other younger child in household	0.99 (0.01 to 1.98)	0.15 (−0.12 to 0.41)
Urban	−0.23 (−1.61 to 1.16)	0.17 (−1.06 to 1.4)
Batoka	0.97 (−0.96 to 2.9)	−2.17 (−3.2 to 1.13)
Choma Railway Surgery	0.26 (−1.01 to 1.52)	0.28 (−0.89 to 1.45)
Kamwanu	4.70 (2.98 to 6.41)	−0.03 (−0.81 to 0.76)
Macha	−1.05 (−2.21 to 0.11)	0.07 (−0.68 to 0.82)
Mangunza	−0.93 (−2.13 to 0.27)	0.06 (−0.69 to 0.82)
Mapanza	−0.84 (−2.2 to 0.51)	1.02 (0.29 to 1.75)
Masuku Mission	−0.89 (−2.12 to 0.35)	0.73 (0.00 to 1.47)
Mochipapa	−0.47 (−1.71 to 0.77)	0.53 (−0.22 to 1.28)
Nalituba	−1.26 (−2.52 to 0.00)	−0.39 (−1.29 to 0.5)
Shampande	−0.49 (−1.73 to 0.76)	−0.11 (−1.28 to 1.05)

DTP, diphtheria-tetanus-pertussis; DTP, diphtheria-tetanus-pertussis.

The results of the geostatistical model of measles zero-dose prevalence were also similar to the univariate analyses. Measles zero-dose prevalence decreased with age (at a slower rate than DTP zero-dose prevalence), while an increase in travel time to the nearest facility and the presence of at least one older eligible child in the household were both associated with increased measles zero-dose prevalence. The posterior mean for the effect of a household being between facilities was also positive, although the 95% credible interval for this coefficient narrowly contained zero. Batoka catchment was associated with lower measles zero-dose prevalence, while Masuku Mission and Mapanza catchments were associated with increased prevalence.

**Figure 4:**
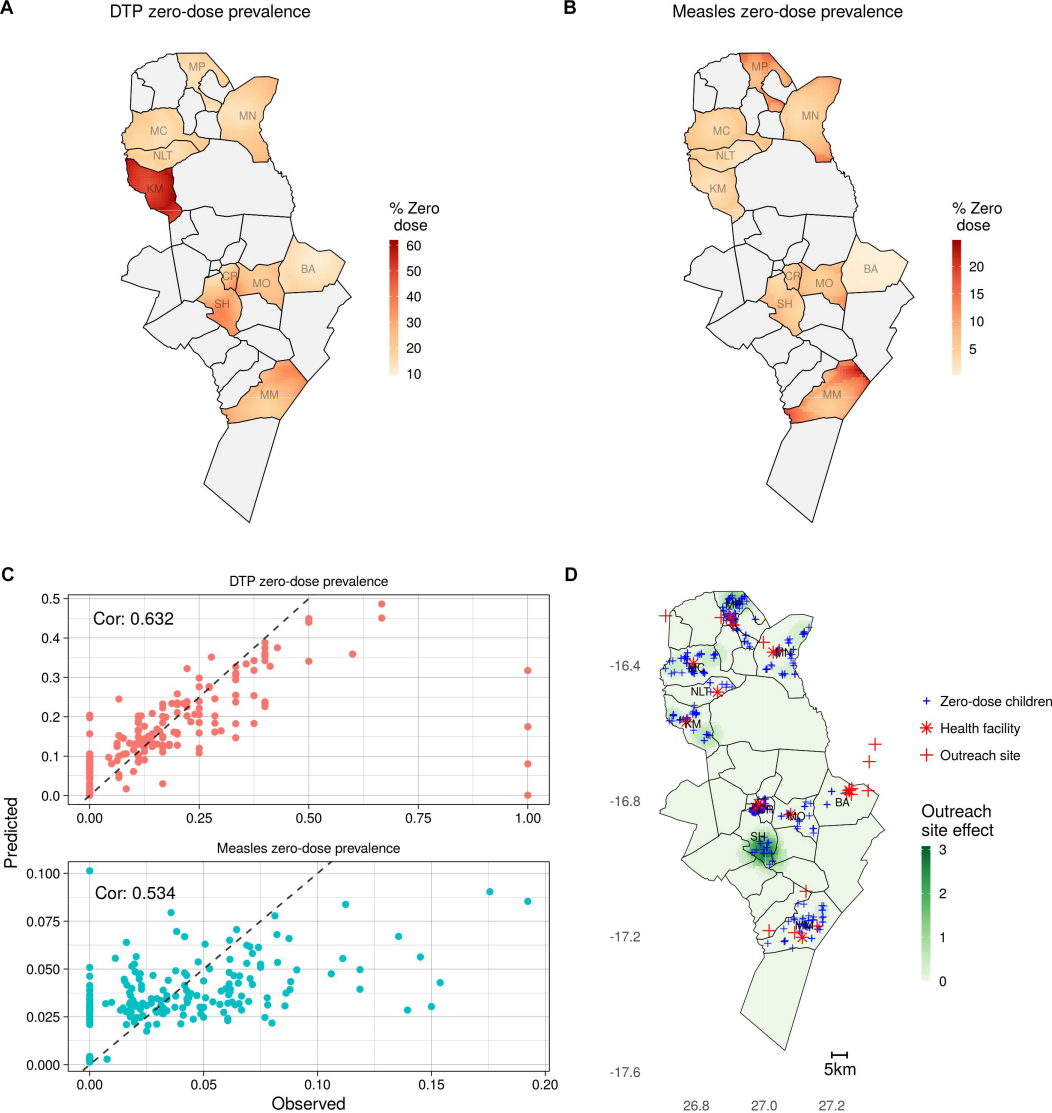
Results from geostatistical models. (A) and (B) Predicted DTP and measles zero-dose prevalence before the mass vaccination campaign, respectively. (C) Predicted and observed DTP and measles zero-dose prevalence during cross-validation at the settlement level. (D) Predicted effectiveness (overall increase in vaccination probability over all measles zero-dose children) of adding an additional vaccination site in each location (shown in green with darker green representing greater effectiveness), with locations of measles zero-dose children shown by blue crosses, health facilities as red stars and current outreach sites as red crosses. DTP, diphtheria-tetanus-pertussis.

Many of the patterns in the maps of predicted zero-dose prevalence (figure 4A, B) reflect the catchment-level patterns in the observed data, such as substantially higher DTP zero-dose prevalence in Kamwanu and lower measles zero-dose prevalence in Batoka. There is also subdistrict heterogeneity apparent in both maps, driven by the spatial covariates relating to health facility access and trends in the observed data learnt by the Gaussian process term in the model. The model performed well under 10-fold cross validation (figure 4C), with correlations between the observed and predicted prevalence at the settlement level of 0.632 and 0.534 for children younger than 9 months and 9 months and older, respectively (see supplementary material section 3 for details of additional validation).

Table 3 shows the fitted coefficient values from the geostatistical model of the probability of a measles zero-dose child being vaccinated during the mass vaccination campaign. Increased distance from a campaign site was associated with decreased probability of vaccination. There was no clear relationship between vaccination during the mass vaccination campaign and age. Magunza and Mochipapa catchment areas were associated with increased probability of vaccination, while Macha catchment was associated with decreased probability. When the alternative form of the model was fit, in which time to the nearest health facility and time to the nearest outreach vaccination site were considered as separate covariates, there was no significant difference in results (see online supplemental material section 3.2).

**Table 3:** Mean and 95% credible interval for covariate effects in the model of probability of a measles zero-dose child being vaccinated during the mass measles and rubella vaccination campaign

Covariate	Mean (CI)
Time to nearest campaign site	−0.52 (−0.74 to 0.30)
Age	−0.10 (−0.31 to 0.11)
Batoka	0.32 (−1.10 to 1.75)
Choma Railway Surgery	−0.77 (−1.63 to 0.09)
Kamwanu	−0.72 (−1.61 to 0.17)
Macha	−1.05 (−1.87 to 0.23)
Mapanza	−0.17 (−0.97 to 0.63)
Mangunza	1.02 (0.01 to 2.02)
Masuku Mission	−0.10 (−0.99 to 0.79)
Mochipapa	1.58 (0.26 to 2.90)
Nalituba	−0.72 (−1.95 to 0.52)
Shampande	0.61 (−0.21 to 1.42)

The results of the geostatistical model of vaccination probability were used to estimate the effect of placing additional vaccination sites in different locations (figure 4D). The greatest estimated impact was in the south of Shampande catchment, in the centre of Choma District, where there were many measles zero-dose children with no campaign site within a 60-min walk. Another area with relatively high impact is in the north of Mapanza catchment, in the north of the district, where again there were measles zero-dose children relatively far from any existing campaign site. There were no areas in the Choma Railway Surgery, Mochipapa and Batoka catchment areas that would have benefited from additional vaccination sites, likely due to already high vaccination probability and low measles zero-dose prevalence in Batoka and Mochipapa or high vaccination site coverage in Choma Railway Surgery.

The optimal locations for three additional sites are shown in online supplemental figure 7 and follow a similar pattern to optimising the effect of a single site, with additional sites in Shampande, Mapanza and Kamwanu. The alternative analysis using measles zero-dose status as negative control found no evidence of confounding (see online supplemental material section 3.3).

## Discussion

Despite substantial progress in expanding vaccination coverage, there remain zero-dose children and communities that are at risk of outbreaks, stalling progress towards disease control and elimination. In areas with high routine vaccination coverage, identifying these communities is necessary to improve targeted and tailored outreach vaccination services. The results of this work highlight substantial fine-scale spatial heterogeneity in the prevalence of zero-dose children in rural Zambia. For both DTP and measles, there was significant variability in vaccination coverage between health facility catchment areas, some evidence of subcatchment clustering of zero-dose children and variation in coverage based on access to healthcare. There was also evidence of fine-scale spatial variation in the effectiveness of the mass measles and rubella vaccination campaign in reaching measles zero-dose children.

This heterogeneity has important implications for disease control, as areas with a high proportion of undervaccinated children may be at risk of outbreaks despite high coverage overall (over 95% of children 9–60 months in the study had received MCV1 before the mass vaccination campaign). The observed increase in zero-dose prevalence as travel time to the nearest health facility increased suggests that travelling long distances may be an impediment to vaccination, as has been previously observed.^14 18 19^ This is also supported by the decrease in proportion of measles zero-dose children vaccinated during the mass vaccination campaign as distance to the nearest vaccination increased. There was also an apparent increase in zero-dose prevalence in areas approximately equidistant from two health facilities and this effect remained for children 9–60 months when controlling for other factors, including travel time to the nearest facility. This may indicate that households in these areas are not being served by either of the nearest health facilities, although there could also be other factors specific to these areas affecting vaccination rates that have not been considered here. The high rates of vaccination during the mop-up activities of measles zero-dose children not reached by the campaign suggests that inadequate access to healthcare or prioritisation may be more likely causes of undervaccination in the study area than vaccine refusal. However, reasons for undervaccination are often complex and multifaceted and more work is needed to understand these reasons in this region, such as qualitative interviews with caregivers of undervaccinated children.

The presence of at least one other eligible child in the household was also associated with increased likelihood of DTP and measles zero-dose status, a similar result to the lower levels of childhood vaccination in larger families found in some studies.^19 21 37 38^ The presence of another child in the household could affect vaccination status directly or this result may reflect correlations between household size and other socioeconomic factors that influence vaccination rates.

Recently the Gavi, global funding agencies, and some national vaccination programme managers have advocated targeted and tailored measles and rubella vaccination activities as opposed to non-selective, nationwide campaigns.^39^ To target intensified periodic routine immunisation activities, it is important to understand the spatial distribution of the zero-dose children or missed communities at a finer, subnational scale. Our analysis of the effectiveness of additional outreach vaccination sites shows how fine-scale spatial data can be used to answer operationally important questions and target allocation of vaccination resources to communities where there are likely to be more zero-dose children. Several areas were identified that had high numbers of measles zero-dose children and were relatively far from existing campaign sites. If outreach vaccination sites were set up in these areas, there is an increased likelihood that measles zero-dose children would be reached, given the relationship between travel time and vaccination. Moreover, by quantifying the effectiveness of the measles and rubella vaccination campaign in reaching measles zero-dose children, we were able to compare the relative impact of adding one or more vaccination sites in different locations. While real-world decision-making regarding planning and targeting of future campaigns will depend on many additional factors, analyses such as these could be a useful starting point for micro planning of routine and campaign-based vaccination activities.

Furthermore, geostatistical models of zero-dose prevalence, such as the ones developed here, could also be used for targeting of household vaccination activities. Our postcampaign household vaccination activities successfully vaccinated all but 14 of the measles zero-dose children initially identified (nine were not reached during follow-up and five were not vaccinated despite being followed up), suggesting that household vaccination could be a highly effective tool for reducing zero-dose prevalence in this area. However, the door-to-door activities carried out in this study before the campaign to identify zero-dose children were labour intensive and expensive. The use of predictive models could remove the need for this exhaustive household enumeration. Instead, limited household data collection in select areas could be performed to gather data to train a geostatistical model. Household vaccination activities could then be targeted to areas where predicted zero-dose prevalence is high. While more work is needed to validate the generalisability and accuracy of these models, such a strategy has the potential to allow for precise targeting of resources to areas of most need in a cost-effective manner.

This work also has implications for the distribution of other vaccines through mass campaigns and for child health programmes more generally. Identifying and successfully providing healthcare to individuals with limited access to or interaction with routine health systems is vital for achieving consistently high coverage of public health interventions. These individuals or communities may be found in areas with high proportions of zero-dose children. Furthermore, other child survival interventions, including child health weeks, use much of the same infrastructure and healthcare providers as the mass measles and rubella vaccination campaign. Therefore, zero-dose children who were not vaccinated during the campaign may also be missed by these interventions. Thus, our approach is applicable to other child health interventions and campaigns.

The household survey strategy described here provided a unique source of information on the fine-scale spatial variation in the prevalence of zero-dose children. The process of identifying structures via satellite imagery enabled data collectors to visit households that were not previously known to the survey team and ensured that remote locations were included. In contrast, routinely collected vaccination coverage data are collected at a more aggregated spatial scale, such as at the health facility or district-level and are therefore likely to mask some of the fine-scale variation. Furthermore, the collection of routine data is likely to be biased towards individuals or communities with better access to the healthcare system and therefore may overestimate coverage. This can be seen in the high coverage values in many districts (often above 100%) where true coverage is believed to be much lower.^24 40^ Another common source of vaccination coverage data is from Demographic and Health Surveys (DHS)^41^; however, these data are unable to provide the same level of spatial granularity as the data analysed here due to privacy-preserving spatial displacement and less dense sampling schemes over much wider areas. In the 2018 DHS in Zambia, for example, there were only four clusters in our study area and eight in Choma District.^23^

One limitation of this study is the limited demographic information collected. While the number of questions on the survey was intentionally limited to reduce the time taken to conduct each survey, more demographic information (such as indicators of socioeconomic status) would have allowed more factors potentially influencing vaccination status to be considered. This could have provided more insights into areas likely to have high numbers of zero-dose children in different districts or in the health facility catchment areas not included in the study. Similarly, conducting the survey in more than one district could have improved the generalisability of these findings and informed the reliability of some of the associations found. We performed several model validation steps and found that the model performed well during testing. However, the model may not perform as well in areas with substantially different geographic, and demographic characteristics than Choma District. Furthermore, we focused our analysis on children identified as DTP or measles zero-dose, defined as those that had not received DTP1 or MCV1, respectively. This did not include children who were partially immunised, that is, those who received MCV1 but not MCV2, who may represent another partially susceptible population. Additionally, data were not collected on DTP1 vaccination status for children 9 months and older, which would have allowed us to compare access to routine services more broadly. It could also have been useful to conduct more detailed interviews with caregivers or heads of households in which zero-dose children were identified regarding reasons for non-vaccination. Finally, our household data collection activities before the mass vaccination campaign may have influenced the coverage of the campaign, as the household data collection activities may have increased awareness in the community of the upcoming campaign. Any such effect would likely have increased vaccine uptake, however, strengthening the implications of our finding that a quarter of measles zero-dose children were not reached during the campaign. The data collection procedures for this study did not influence how the mass vaccination campaign was carried out, as the campaign was conducted following the traditional approach used by the health centres and did not use our data to make decisions on location of fixed and outreach posts.

## Conclusion

We provide one of the first spatial analyses of the prevalence of zero-dose children and possible strategies to improve targeted and tailored measles and rubella vaccination activities. Our analysis indicates that greater travel time to healthcare (either health facilities or vaccination sites) was associated with increased zero-dose prevalence and decreased likelihood of a measles zero-dose child being vaccinated in the mass vaccination campaign. The presence of other children in the household and living approximately equidistant from two health facilities were also associated with greater zero-dose prevalence. The fine-scale spatial variation in zero-dose prevalence highlights the potential benefits of subdistrict targeting and microplanning of vaccination activities and child health services more broadly.

## Data Availability

Data are available upon reasonable request. Restrictions apply to the availability of these data. Data were obtained under data sharing agreements from Zambia Ministry of Health and the Zambia National Health Research Authority and can only be shared with their permission from the Ministry of Health.
